# Neonatal encephalopathy and hypoxic-ischemic encephalopathy: the state of the art

**DOI:** 10.1038/s41390-025-03986-2

**Published:** 2025-03-24

**Authors:** Ela Chakkarapani, Linda S. de Vries, Donna M. Ferriero, Alistair J. Gunn

**Affiliations:** 1 Population Health Sciences, Bristol Medical School, University of Bristol, Bristol, UK; 2 Department of Neonatology, Leiden University Medical Center, Leiden, The Netherlands; 3 Department of Neonatology, University Medical Center Utrecht, Utrecht, The Netherlands; 4 Departments of Neurology and Pediatrics, University of California, San Francisco, CA USA; 5 Department of Physiology, University of Auckland, Auckland, New Zealand

## Abstract

**Abstract:**

Neonatal Encephalopathy (NE) remains a major cause of death and long-term severe disabilities, including epilepsy and cerebral palsy in term and near-term infants. The single most common cause is hypoxic-ischemic encephalopathy (HIE). However, there are many other potential causes, including infection, intracranial hemorrhage, stroke, brain malformations, metabolic disorders, and genetic causes. The appropriate management depends on both the specific cause and the stage of evolution of injury. Key tools to expand our understanding of the timing and causes of NE include aEEG, or even better, video EEG monitoring, neuro-imaging including cranial ultrasound and MRI, placental investigations, metabolic, biomarker, and genetic studies. This information is critical to better understand the underlying causes of NE. Therapeutic hypothermia improves outcomes after HIE, but there is still considerable potential to do better. Careful clinical and pre-clinical studies are needed to develop novel therapeutics and to help provide the right treatment at the right time for this high-risk population.

**Impact:**

- Neonatal encephalopathy is complex and multifactorial.
- This review seeks to expand understanding of the causes, timing, and evolution of encephalopathy in newborns.
- We highlight key unanswered questions about neonatal encephalopathy.

## Introduction

Neonatal encephalopathy (NE) in term and near-term infants is defined as altered neurologic function in a newborn infant, characterized by altered consciousness and abnormal tone and reflexes. This broad definition does not specify etiology, or guide diagnosis and management, and the pragmatic definitions vary widely between studies. The single most common cause of NE is hypoxic-ischemic encephalopathy (HIE) but it can also be associated with infection, intracranial hemorrhage, stroke, brain malformations, metabolic disorders, genetic causes, and combinations thereof. This broad differential must be considered and carefully scrutinized to make the correct diagnosis and inform management and outcomes. NE and HIE have often been used interchangeably, leading to confusion around root causality. Here we will describe many etiologies of NE and ways of approaching the diagnosis of specific types of NE, focusing on recent updates and future research directions.

## The current approach to the diagnosis of neonatal encephalopathy

A detailed review of the pregnancy, maternal medical and family history, and of labor and delivery should precede evaluation of the infant. However, given that newborn infants with encephalopathy must be considered for therapeutic hypothermia (TH) within 6 h of birth,^1^ it is not always immediately clear whether NE is due to HIE or whether another diagnosis might be involved. A focused history and examination to diagnose HIE is recommended. Perinatal sentinel events (e.g.,: placental abruption, shoulder dystocia, prolapse of the umbilical cord), fetal heart rate abnormalities consistent with exposure to hypoxemia in labor, need for resuscitation at birth, severe acidosis in the perinatal blood gas (cord blood pH <7.0 or base excess > 15 mmol/L), Apgar ≤5 at 10 min after birth and being encephalopathic soon after birth suggest hypoxia-ischemia (HI) as the likely cause for the neonatal encephalopathy, although it can still occur with less severe acidosis.^2^ The presence and severity of encephalopathy must be confirmed by clinical neurological exam (e.g., Sarnat neuro exam or Thompson score), ideally supported by electroencephalographic (EEG) or amplitude integrated EEG (aEEG) evidence of abnormal brain activity. Further evidence to support the diagnosis of moderate to severe HIE can include multi-organ impairment involving the liver (raised transaminases), kidney (oliguria, elevated creatinine), thrombocytopenia, or coagulopathy, although it is important to appreciate that it is possible to have HIE without systemic injury.^3^ HIE can be associated with acute or subacute or chronic placental lesions; however, pathology is typically only available in retrospect, and even if such lesions are present, at present they are neither necessary nor sufficient for a diagnosis of HIE.^4^ Further, even if HIE is confirmed, in principle it is still possible for additional factors to contribute to encephalopathy.^5^

Other factors contributing to NE can include maternal infection, medication or substance use, hypertensive disorders (pregnancy-induced hypertension, preeclampsia, chronic hypertension or HELLP syndrome), maternal obesity, birth trauma and abnormalities on antenatal ultrasound, or history of recurrent miscarriages or consanguinity or a family history of genetic disorders, neonatal illness, seizures, cerebral palsy or learning difficulties and importantly delayed onset of encephalopathy after birth.^6,7^ Specific causes of NE are described in the body of this review.

## Search strategy and selection criteria

We searched Pubmed and the Cochrane Database of Systematic Reviews (CDSR) for publications in English from Jan 1, 2019, to July 1, 2024, using the search terms: Neonatal encephalopathy OR hypoxic ischemic encephalopathy OR (neonatal seizures AND encephalopathy) OR neonatal infection OR neonatal cerebral hemorrhage OR neonatal stroke AND epidemiology OR treatment OR neurodevelopmental follow-up OR magnetic resonance imaging OR (metabolic disorders OR inborn errors of metabolism including dysglycemia) OR genetic etiologies. Additional references were identified by searching the references in randomized controlled trials for NE.

## Hypoxic-ischemic encephalopathy

Metabolic acidosis (pH < 7.2) at birth is an important, dose-related risk factor for neonatal outcomes in general, presumably as a marker of the extent of anaerobic metabolism.^8^ In many studies, severe acidosis with cord blood pH < 7.00 was used to help select infants at risk of HIE.^9^ However, in a recent population-based cohort study of 340 431 infants born from 2012 to 2018 in Denmark,^10^ severe adverse neonatal outcomes (including death, seizures, need for therapeutic hypothermia (TH), need for mechanical ventilation, and hypoglycemia) increased progressively compared to infant without acidosis as the umbilical pH fell below 7.2; 7.10 to 7.19 (259 of 73,244 (0.4%)), 7.00 to 7.09 (101 of 11 904 (0.8%)) and < 7.00 (171 of 1,743 (9.8%)), similarly to a previous meta-analysis.^8^ Neonatal death was only increased if the pH was < 7.10. Consistent with this, in a cohort study of 27028 infants, Vesoulis et al. found that although the rate of moderate to severe encephalopathy was highest for infants with cord blood pH <7.00 (35/85), there was a still significant risk of HIE in infants with pH 7.00–7.10 (34/327).^2^ These important studies suggest that our current threshold of acidosis for intensive observation and treatment is too low.

Originally, both HIE and its severity were defined in retrospect, based on evidence of a metabolic acidosis or need for resuscitation, combined with evolving encephalopathy based on clinical examination and aEEG/EEG and imaging, as detailed in Tables 1–3.^11^ Since TH became standard care in high-income countries, it is now essential to identify probable HIE as early as possible within the first 6 h of life in order to initiate treatment at a time when it is likely to be effective. Thus, most studies now use a staged combination of evidence of perinatal events followed by a modified Sarnat score and/or acute aEEG/EEG examination. Pragmatically, this approach does not seem to be associated with significant misdiagnosis. In a recent large trial, only 4% of infants had an additional neurological diagnosis that likely increased their ultimate level of disability.^12^ Interestingly, 5% had either a genetic diagnosis or a congenital abnormality. Thus, misdiagnosis is unlikely to substantially contribute to the risk of disability despite TH.

**Table 1:** Definitions of hypoxic-ischemic encephalopathy after perinatal asphyxia used in randomized controlled trials of therapeutic hypothermia.

Category	Clinical trials				
	CoolCap^136^	NICHD^137^	TOBY^138^	ICE^139^	neo.nEuro^140^
Gestation	≥ 36 weeks	≥ 36 weeks	≥ 36 weeks	≥ 35 weeks	≥ 36 weeks
Peripartum asphyxia Cord or blood gas within 60 min of birth Apgar score at 10 min after birth Resuscitation at 10 min after birth	pH < 7.0 (or) base deficit (BD) ≥ 16 mmol/L (or) ≤ 5 (and) Endotracheal or mask ventilation AND	pH ≤ 7.0 (or) BD ≥ 16 mmol/L if pH:7.01–7.15; BD:10–15.9; or no blood gas, then ≤ 5 (or) Assisted ventilation (and) Acute perinatal event AND	pH < 7.0 (or) BD ≥ 16 mmol/L (or) ≤ 5 (or) Need for resuscitation AND	At least 2 of the following pH < 7.0 (or) BD ≥ 12 mmol/L (or) ≤ 5 (or) Mechanical ventilation AND	≥ 1 criteria pH < 7.0 (or) BD > 16 mmol/L (or) <5 (or) Endotracheal intubation or mask ventilation AND
Neurology	Lethargy, stupor, or coma + 1. hypotonia (or) 2. abnormal reflexes (including oculomotor or pupil abnormalities) (or) 3. absent or weak suck (or) 4. clinical seizures AND	3/6 abnormalities in 1. Level of consciousness 2. Spontaneous activity 3. Posture 4. Tone 5. Primitive reflex 6. Autonomic system (or) Clinical seizures	Lethargy, stupor, or coma + 1. hypotonia (or) 2. abnormal reflexes (including oculomotor or pupil abnormalities) (or) 3. absent or weak suck (or) 4. clinical seizures AND	Lethargy Stupor Coma Abnormal tone Seizures	Lethargy, stupor, or coma + 1.hypotonia (or) 2.abnormal reflexes (including oculomotor or pupil abnormalities) (or) 3.absent or weak suck (or) 4. clinical seizures AND
aEEG	20 mins duration, 1 h after birth Moderately or severely abnormal	Not used	30 mins duration Moderately or severely abnormal	Not used	Moderately or severely abnormal
Cooling commenced	Within 6 h after birth	Within 6 h after birth	Within 6 h after birth	Within 6 h after birth	Within 6 h after birth
Target temperature	Rectal: 34–35 °C	Esophageal: 33.5 °C	Rectal: 33–34 °C	Rectal: 33–34 °C	Rectal: 33–34 °C
Duration of cooling	72 h	72 h	72 h	72 h	72 h
Mode of cooling	Selective head cooling (coolcap + radiant warmer)	Whole body cooling (servocontrol)	Whole body cooling (manual mode)	Whole body cooling (gel packs + radiant warmer)	Whole body cooling (manual mode)

**Table 2:** Definition of hypoxic-ischemic encephalopathy in trials of therapeutic hypothermia in LMICs.

Category	Akisu^141^	Lin^142^	Zhou^143^	Robertson^144^	HELIX trial^71^
Countries	Turkey	China	China	Uganda	India, Sri Lanka and Bangladesh
Gestation	>37 weeks	≥ 37 weeks	≥ 37 weeks	≥ 37 weeks	≥ 36 weeks
Gross National income	Upper middle	Upper middle	Upper middle	Low	Lower middle
Age	Soon after birth	<6 h after birth	<6 h after birth		≤ 6 h after birth
Asphyxia	5 min Apgar <6 (and) Cord pH <7.1 or BD > 10 mmol/L AND	5 min Apgar <6 (and) Cord pH <7.1 or BD > 15 mmol/L AND	5 min Apgar <6 (and) Cord pH<7 or BD ≥ 16 mmol/L (and) Resuscitation at 10 mins of age AND	Need for resuscitation (and / or) 5 min Apgar <6 AND	Infants born in hospital Continued resuscitation at 5 mins after birth (or) (and) Apgar <6 at 5 mins after birth Infants born at home Absence of crying by 5 mins after birth AND
Encephalopathy	stupor hypotonia abnormal neonatal reflexes	decreased muscle tone lethargy coma (or) seizures	Lethargy, stupor or coma + hypotonia (or) abnormal reflexes (or) clinical seizure	Thompson score >5 from 30 min to 3 h after birth	3/6 abnormalities in modified Sarnat exam 1.Level of consciousness 2. Spontaneous activity 3.Posture 4.Tone 5.Primitive reflex 6.Autonomic system (or) Clinical seizures
Target temperature	Auditory canal temp: 33.0–33.5 °C	Rectal: 34–35 °C	Rectal: 34.5–35 °C	Rectal: 33–34 °C	Rectal: 33.5 °C
Duration	72 h	72 h	72 h	72 h	72 h
Mode of Cooling	Selective head cooling	Selective head cooling	Selective head cooling	Whole body cooling (water bottles)	Whole body cooling Servo-controlled

**Table 3:** Definitions of hypoxic-ischemic encephalopathy in studies in the era of therapeutic hypothermia in high income countries.

Category	Weeke^145^	Thoresen^146^	HEAL^12^
Countries	Netherlands, Sweden	UK	US
Gestation	> 36 weeks	≥ 36 weeks	≥ 36 weeks
Asphyxia	5 min Apgar ≤5 pH ≤7.10 BD ≥ 16 mmol/L (or) Resuscitation at 10 min after birth	At least one of pH within 1 h after birth <7.0 BD within 1 h after birth ≥ 16 mmol/L 10 min Apgar ≤5 Need for assisted ventilation at 10 min after birth	≥ 1 criteria 10 min Apgar <5 (or) Resuscitation >10 min of age (or) pH within 1 h after birth <7.0 (or) BD within 1 h after birth ≥ 15 mmol/L
Encephalopathy	Thompson score >7 (and/or)	Lethargy, stupor or coma plus 1.hypotonia (or) 2.abnormal reflexes (including oculomotor or pupil abnormalities) (or) 3.absent or weak suck (or) 4.clinical seizures AND	3/6 Sarnat criteria Reduced consciousness Decreased spontaneous activity Hypotonia Decreased suck reflex Decreased Moro reflex Respiratory abnormality
aEEG	Discontinuous EEG	Moderate to severely abnormal aEEG	
Additional diagnoses		Postnatal collapse	

## Epidemiology of hypoxic-ischemic encephalopathy

In high-income countries, studies using a consistent definition of HIE over time reported a fall in the incidence of moderate to severe HIE from 1–5/1000 live births in the early 2000s to 0.5–1.9/1000 livebirths in 2008–2010, which was attributed to improved antenatal and perinatal care.^13^ More recently, a population-based cross-sectional study in the US of hospital live births of infants born ≥ 35 weeks’ gestation age, found a stable incidence of perinatal HIE of 1.7 per 1000 live births between 2012 and 2019.^14^ By contrast, in low- and middle-income countries (LMICs) the incidence of HIE remains high, ranging between 1.5 to 20.3/1000 live births, with more severe encephalopathy and a higher case fatality rate.^15^

## Patterns of HIE on modern imaging

Magnetic resonance imaging (MRI) is the recommended neuro-imaging technique for infants with neonatal encephalopathy (NE), including infants treated with hypothermia.^16^ MRI is recommended for infants with probable HIE, particularly in the presence of seizures. In more than 90% of neonates admitted with NE and seizures, MRI showed abnormalities suggestive of a diagnosis.^17^ For all infants presenting with NE the use of T1- and T2-weighted sequences, diffusion-weighted imaging (DWI), and susceptibility weighted imaging (SWI) is recommended. Slice thickness should be 2 mm or less. When arterial ischemic stroke or sinovenous thrombosis is suspected, MR angiography and MR venography should be added to the protocol. ^1^H-MR spectroscopy will provide very useful information and should be performed when available.^17^ Advanced imaging techniques, such as DTI, TBSS, arterial spin labeling (ASL), and the use of machine learning significantly improve prediction, at least in a research setting.^18^ The best time to perform the MRI in an infant treated with TH using diffusion weighted imaging (DWI) is shortly after rewarming (day 4–6). This will allow more reliable identification of acute injury especially in the central grey nuclei.^19^ Performing the MRI during TH will interrupt cooling and may not show the full extent of the injury but may be helpful when redirection of care is considered.^20^ Pseudo-normalization of DWI occurs by the end of the first week.^21^ When the MRI is performed during the second week of life, signal intensity changes will have become clear on T1- and T2 weighted sequences. Meta-analysis suggests that the diagnostic odds ratio (DOR = (true positives/false positives)/(false negatives/true negatives)) is greater when the MRI was performed during the first rather than the second week of life.^22^

**Fig. 1:**
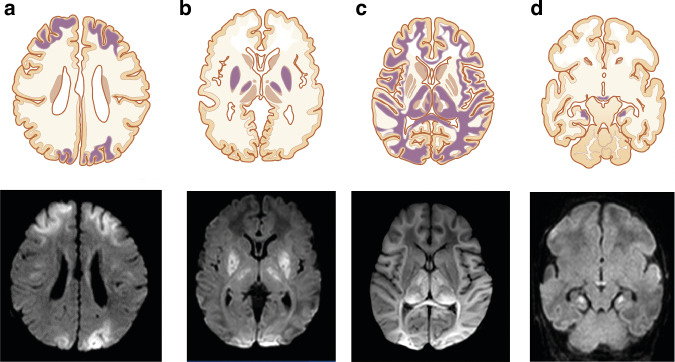
Schematic drawings (top row) and axial MRI-diffusion weighted images taken during the first week of life (bottom row) showing common patterns of brain injury seen in infants with hypoxic-ischemic encephalopathy. From left to right: bilateral watershed injury (**a**); basal ganglia/thalamic injury (**b**); near total pattern of injury (**c**) and injury to the mammillary bodies and hippocampi (**d**).

The use of a scoring system is recommended to support systematic assessment of the presence and extent of the main patterns of injury. 1) predominant injury in the basal ganglia and thalami (BGT), mainly the ventrolateral thalami and posterior putamina, and the perirolandic cortex following acute profound asphyxia; 2) injury in the parasagittal cortex and the subcortical white matter following partial prolonged asphyxia (watershed/white matter injury) and 3) diffuse injury in both the BGT and white matter (near total pattern) following very severe and prolonged asphyxia (Fig. 1). Several scoring systems have been introduced since the Barkovich score.^23^ The Rutherford score, Trivedi score, Weeke score, and NICHD NRN score are reported to have similar area under the curve (AUC > 0.66) for adverse outcome^24^; of these the Weeke and Trivedi scores had the highest inter-rater reliability.

By contrast, in a retrospective cohort study of 162 infants with probable HIE who were treated with TH, the AUC for ROC analysis for composite adverse outcome of death or moderate or severely delayed development at 2 years of age was significantly better for the Weeke score, 0.81 (95% CI 0.74–0.88), than the Barkovich score, 0.73 (95% CI 0.66–0.81).^25^ Further, the Weeke score was reported to provide better prediction of language score at two years than the Barkovich or NICHD systems (adjusted R^2^ = 0.166, *P* = 0.005).^26^ The Trivedi and Weeke scoring systems include DWI plus abnormalities in the cerebellum and brainstem. The Weeke score also includes intraventricular, extra-axial hemorrhages, sinovenous thrombosis, and ^1^H-MRS. Because it includes more detailed information the Weeke score may be especially useful for infants with mild HIE^27^; further studies are needed to validate this suggestion.

While moderate to severe injury to the central grey nuclei is associated with poor motor outcome, watershed injury is more likely to be associated with adverse cognitive outcomes, which may not be apparent until the children are seen at school age and seem to be more clearly defined in early adolescence.^28^ Another brain structure that may be a potential biomarker of cognitive outcome is the mammillary body. These are part of the limbic system, are small, and can only be assessed on 2 mm slices. An increase in signal intensity with swelling is best seen on the T2 sequence or with restricted diffusion. Mammillary body involvement can be an isolated finding, with or without associated hippocampal injury, or other brain injury. Within 4–6 weeks, the MRI will show atrophy of these structures, which is best seen on the sagittal T1 or T2 sequence.^29^

Quantitative MRI-based apparent diffusion coefficient (ADC) metrics for the whole brain and within the thalami and white matter, were recently reported in a secondary analysis of 416 of 500 infants recruited to the HEAL trial, based on automatically measuring areas with ADC values less than 800 × 10^−6^ mm^2^/s.^30^ A total volume of reduced ADC > 1 ml was associated with an increased odds ratio (OR 13.9; CI 5.93–32.45) for death and neurodevelopmental impairment at 2 years of age than the presence of any acute neural injury (OR 4.5; CI 2.6–7.8). Other advanced MRI techniques, such as arterial spin labeling (ASL) and diffusion data analyzed using frameworks such as tract based statistical analysis (TBSS) may also be helpful in predicting outcomes.^31,32^

When available, proton magnetic resonance spectroscopy (^1^H-MRS) can provide additional prognostic insight. MRS measures the signal from protons in different chemical metabolites, the main ones being choline (Cho), Creatine (Cr), N-acetylaspartate (NAA). With HI injury, NAA declines over days to weeks and then remains low. In contrast, lactate peaks during the first few days after acute brain injury and then declines toward “normal” by the end of the first week of life.^33,34^ Comparing the predictive value of clinical assessment, aEEG, MRI including DTI and MRS, Lac-NAA ratio (AUC 0.94 (95% CI 0.89–0.97) and NAA concentration (AUC 0.99, 95% CI 0.94—1.00) had the highest AUC.^34^ In another study MRS was performed at 24–96 h and day 7–14 of life, total NAA concentration in the central grey nuclei had the highest prognostic value at both time points (AUC at 24–97 h 0.97 and at 7–14 days 1.00). ADC values (AUC 0.90; 95% CI 0.64–0.98) and lactate-NAA ratios (0.97; 95% CI 0.90–0.99) also had high prognostic values on the early scan.^35^

## Neuromonitoring (aEEG, EEG, NIRS)

While neuro-imaging techniques will provide important information, they are not informative within the first 24 h of life, as the echogenicity that can be seen on cranial ultrasound will take 24–48 h to develop, and although DWI -MRI will already show some abnormalities, retrospective studies show that they do not reach their full extent at that time.^36^ Neurophysiology, including aEEG and continuous video multichannel EEG (cEEG) are increasingly used immediately after admission and may also be of value when deciding whether or not to use TH. In most centers, aEEG is immediately available. It can reliably assess background activity but is less reliable for identifying seizures. Having a multichannel EEG with display of 2 aEEG channels will allow beside monitoring of the aEEG with simultaneous access to the raw EEG, while the neurophysiologist will be able to assess the multichannel EEG at regular time-points and on request. Both aEEG and cEEG are reliable predictors of neurodevelopmental outcomes. The greatest prognostic value of aEEG/cEEG background in those receiving TH is later than for neonates who do not receive TH, with the greatest prognostic value of aEEG/EEG recording around 48–72 h after birth.^37^ Seizures are seen in about one-third of infants who receive TH, often within 12–24 h after birth. Although the presence of seizures per se does not predict outcomes, the magnitude of the seizure burden has been independently associated with adverse outcomes.^38,39^

Seizures can be seen during and after rewarming, more often in infants with a worse EEG background and seizures during hypothermia. aEEG/cEEG monitoring should therefore be performed during hypothermia and continued during rewarming.^40,41^ Interestingly, a small trial of continuous EEG monitoring in 35 infants randomized to treatment of electrographic seizures or clinical seizures only, showed that treatment of electrographic seizures reduced seizure burden,^42^ and although the treatment groups did not appear to have different outcomes, seizure burden in the combined group was associated with both MRI injury and neurodevelopment outcome. Large RCTs are needed to resolve whether seizure treatment improves long-term outcomes.

Near infrared spectroscopy (NIRS) is a non-invasive, bedside monitoring system that continuously measures regional cerebral tissue oxygenation (rScO2). Several studies have reported an increase in rScO2 between day 1 and 2 in infants with an abnormal neonatal MRI and an adverse outcome.^43^ This association of increased rScO2 with brain injury/poor outcome likely reflects impaired mitochondrial oxidative metabolism and luxury perfusion.

## Management of HIE

**Fig. 2:**
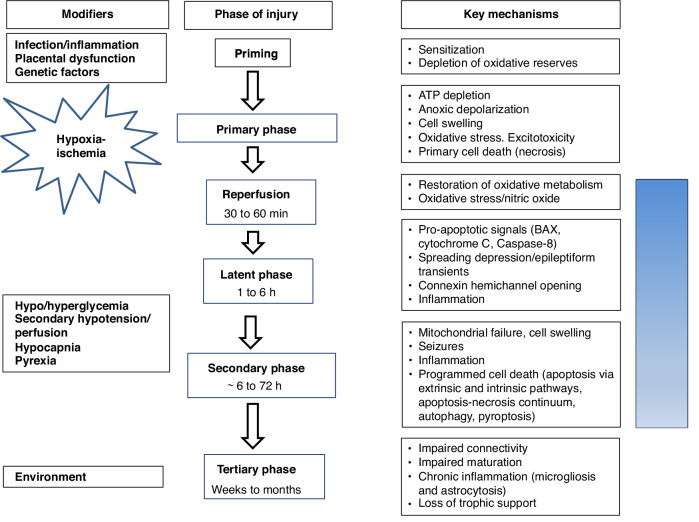
Schema showing the time course of the pathophysiological phases of injury, key mechanisms, and modifying factors before and after hypoxia-ischemia. The blue bar shows when therapeutic hypothermia needs to be applied to achieve effective neuroprotection.

Foundation studies established that the brain often shows partial or even complete recovery of energy metabolism after acute HI in a short “latent” phase, typically lasting ~6 h, only to decline hours to days later, during a “secondary” deterioration phase characterized by seizures, cytotoxic edema and mitochondrial failure (Fig. 2).^44^ Critically, translational studies then showed that by inducing mild hypothermia as soon as possible during the latent phase and continuing it for 72 h it was possible to dramatically improve histological and functional outcomes.

## Therapeutic hypothermia

Meta-analysis of subsequent large randomized controlled trials (RCTs) in high income countries (HICs) confirmed that within strict parameters TH significantly improved survival without disability after moderate-to-severe HIE, with a number needed to treat for each survivor without disability of 8 (95% CI 5 to 14).^45^ Based on this evidence, TH is now part of standard care for HIE in high-income countries,^46^ and remains the only proven treatment for HIE.^11^

Subsequent studies have focused on trying to further improve outcomes and to assess groups who were not included in the original RCTs. In infants with moderate or severe HIE, delayed initiation of TH at 6–24 h after birth and continued for 96 h, demonstrated no significant effect on death or disability.^47^ There was a 76% posterior probability (adjusted posterior risk ratio, 0.86; 95% credible interval, 0.58–1.29) of reduced death or disability compared with non-cooled infants, using Bayesian analysis assuming a neutral prior.^47^ However, the probability of death or disability in cooled (0.244) and non-cooled group (0.278) was too close to be certain of benefit of initiating cooling 6 h after birth.^48^ In a large RCT (*n* = 364), prolonged (120 h) or deeper (32.0°C) hypothermia did not reduce the risk of death or moderate or severe disability compared with the standard regimen of TH to 33.5 °C for 72 h.^49^ Death or moderate or severe disability occurred in 29.3% in the 33.5 °C for 72 h group; 34.5% in the 32.0 °C for 72 h group; 34.4% in the 33.5 °C for 120 h group and 28.2% in the 32.0 °C for 120 h group. It is important to note that although there was no overall effect, the adjusted relative risk for death was higher in the group cooled to 33.5°C for 120 h compared with 33.5°C for 72 h (RR: 2.52; 95% CI, 1.06–5.95).

Infants with mild HIE were excluded from the initial RCTs of the TH. However, meta-analysis of 20 studies found adverse outcomes in 86/341 (25%) infants with mild HIE.^50^ It is still unknown whether infants with mild HIE would benefit from TH. A recent RCT of 101 infants found no significant effect on cerebral magnetic resonance indices of outcome. However, there was evidence of a structural bias, such that infants treated with TH had greater severity of illness, including greater need for invasive resuscitation at birth.^51^ Large, well-designed trials will be important to determine whether infants with mild HIE should receive TH. At present two trials are enrolling, the COMET RCT and the COOL-PRIME comparative effectiveness trial.^52,53^

## Lack of benefit with adjunct therapies during therapeutic hypothermia

The endogenous growth factor erythropoietin is neuroprotective as monotherapy after HI in multiple species and paradigms, provided that it is started within the first few hours of the latent phase.^54,55^ However, there is conflicting evidence for whether it can augment TH.^54,56^ Meta-analysis of 3 small studies in LMICs suggested that erythropoietin monotherapy reduced death or disability from 49.7% to 27.6% in the erythropoietin group (risk ratio 0.56 (95% CI: 0.42–0.75)).^57^ This encouraging finding supports large, well-designed studies to evaluate this approach for infants in whom TH is not appropriate.

By contrast, phase II and III studies of adjunct therapies for TH suggest lack of further benefit. In the High-Dose Erythropoietin For Asphyxia And Encephalopathy (HEAL) trial of 500 infants receiving TH for moderate or severe HIE, 5 doses of 1000 u/Kg erythropoietin on day 1, 2, 3, 4 and 7 days of age did not reduce the risk of death or neurodevelopment impairment compared to placebo, RR:1.03; 95% CI, 0.86 to 1.24, and increased the risk of having a high externalizing behavior score, RR:4.86, 95% CI,1.46 to 16.17.^12^ Moreover, the erythropoietin plus TH group had slightly higher mean number of serious adverse events per child than the placebo group (RR: 1.26; 95% CI, .01 to 1.57), and of at least one serious adverse event (RR:1.21; 95% CI 1.00 to 1.45).

Further, in a phase 2 study (*n* = 92), inhalation of 30% xenon for 24 h commenced at 10 h of age in infants receiving TH compared to TH alone did not reduce the Lactate/NAA ratio in the thalamus (mean (SD) 0.68(1.12) vs 0.47(0.94)) or preserve fractional anisotropy in the posterior limb of the internal capsule (mean (SD) 0.40 (0.01) vs 0.41 (0.01) or death (26.08% vs 21.74%) or moderate or severe disability (25% vs 23%).^58,59^ Relatively delayed start time of treatment (within 26 h after birth) and intermittent dosing may have contributed to the lack of neuroprotection in these clinical trials. Many other potential add-on therapies are being evaluated, such as melatonin and stem cells, but so far there is only preliminary evidence from small studies, as previously reviewed.^60^

## Outcomes after therapeutic hypothermia at school-age

Although children at school age (6–8 years), who underwent TH for neonatal HIE had significantly improved outcomes, many still have motor or cognitive problems. At school age, the relative risk of CP in children cooled for HIE compared with non-cooled children was between 0.59 to 0.6 with 95% CI ranging between 0.31 to 1.18.^61^ All children with CP had IQ < 70, and 9% of children without CP had an IQ < 70 and 31% had an IQ of 70 to 84.^62^ Compared to typically developing children, even children without CP had borderline to extremely low IQs, borderline motor impairment, poorer communication, attention, visuospatial skills and executive function, and behavioral problems^63,64^ and were reported to be less ready for school.^65^

**Fig. 3:**
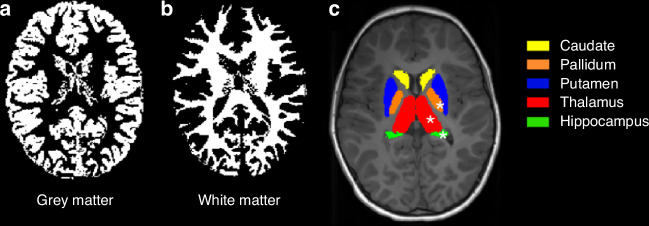
Grey and white matter volumes at school age in children who received therapeutic hypothermia for hypoxic-ischemic encephalopathy at birth. Grey matter volume (**a**); white matter volume (**b**) and subcortical grey matter volumes (**c**). Children cooled for HIE who did not develop CP had significantly lower grey matter (**a**) and white matter (**b**) volumes than healthy controls. Although the thalami, hippocampi, and pallidi volumes (**c**) (asterisk) were lower in children cooled for HIE than controls, they were not independent of total brain volume. Adapted from,^66^ Creative Commons Attribution License.

**Fig. 4:**
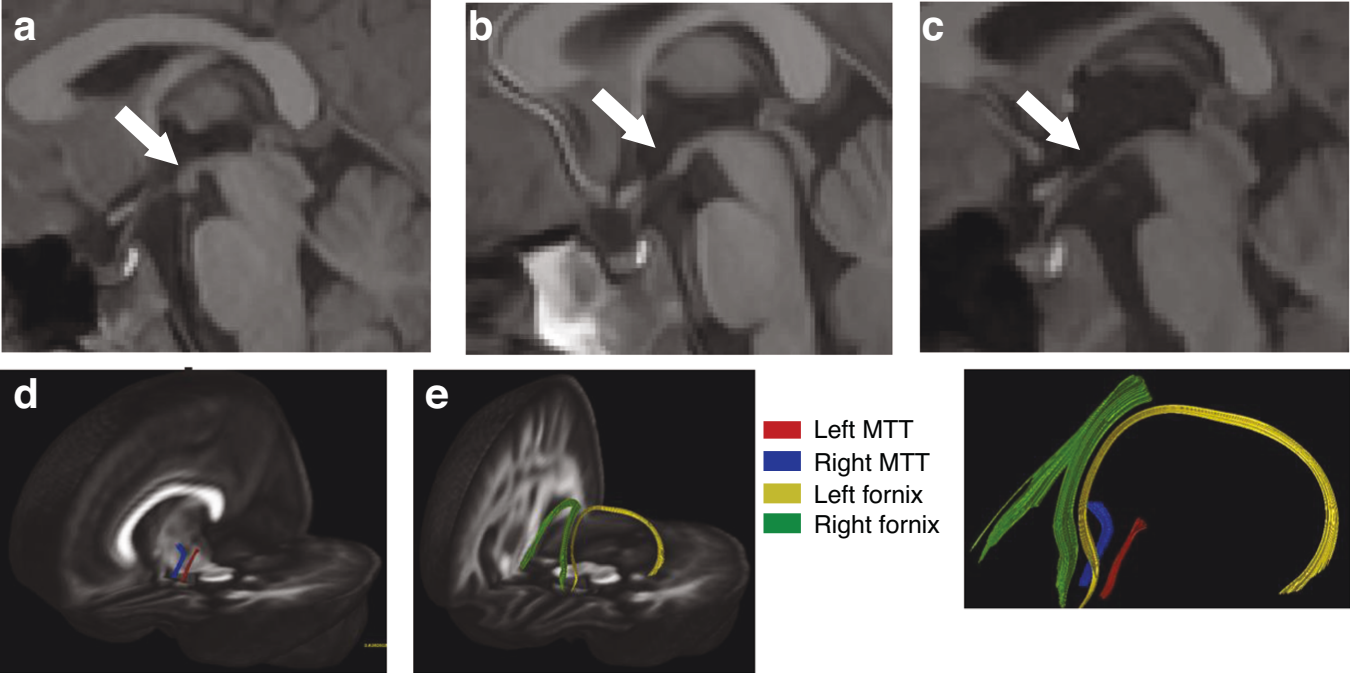
Mammillary body scoring on the sagittal plane of T1 weighted images. Normal (**a**), equivocal (**b**), and abnormal (**c**). Left and right Mammillothalamic tract (MTT) (**d**) and fornix (**e**) shown. Right MTT had increased radial diffusivity in children cooled for HIE (cases) with abnormal mammillary bodies than cases with normal or equivocal mammillary bodies and higher than controls. Left fornix had lower fractional anisotropy in cases with abnormal mammillary bodies than controls. Adapted from ref. ^68^. Creative Commons Attribution License.

On MRI, children cooled for HIE had significantly smaller white matter and grey matter volumes, hippocampi, thalami, and globus pallidi volumes than healthy controls (Fig. 3).^66^ Abnormal mammillary bodies were reported in 35% of children at 6–8 years cooled for HIE and 38% of children cooled and not cooled for HIE at 10 years of age.^67^ Children cooled for HIE who had abnormal mammillary bodies on imaging also had decreased fractional anisotropy in the left fornix and reduced hippocampi volumes (Fig. 4).^68^ Both thalamic and hippocampi volumes were associated with full scale IQ and total motor scores after adjusting for sex, age, and total brain volume in children cooled for HIE but not in controls.^66^

**Fig. 5:**
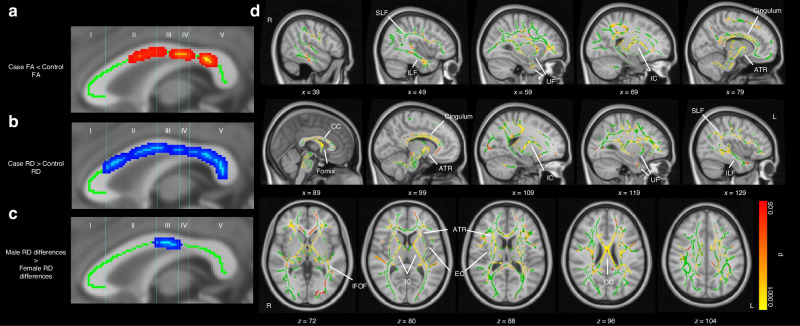
Changes in the microstructure of white matter tracts in children at school age who received therapeutic hypothermia for hypoxic-ischemic encephalopathy in infancy. Decreased Fractional anisotropy in regions of corpus callosum in children cooled for HIE (cases) compared to controls (**a**), areas of increased RD in case children (**b**) and areas in which males exhibit larger case-control differences in RD than females (**c**). Results of voxel-wise comparison of FA on the white matter skeleton (green) showing widespread reduction in FA in children cooled for HIE and did not have CP compared with control children after adjusting for age and sex (**d**). The color bar shows significant differences (*p* < 0.05, Threshold free cluster enhancement corrected. These are overlaid on the MNI standard template. Labels indicate some major white matter tracts and regions. Abbreviations are as follows: anterior thalamic radiation (ATR), corpus callosum (CC), external capsule (EC), internal capsule (IC), inferior fronto-occipital fasciculus (IFOF), inferior longitudinal fasciculus (ILF), superior longitudinal fasciculus (SLF), uncinate fasciculus (UF). Differences are most notable in the IC, CC, fornix, and cingulum. Adapted from.^69,70^ Creative Commons Attribution License.

Further, children cooled for HIE compared to healthy controls have reduced corpus callosum area, volume, fractional anisotropy, and increased radial diffusivity,^69^ with reduced regional fractional anisotropy (Fig. 5),^70^ and reduced cognitive scores compared with healthy controls, with significant correlation between the magnitude of changes and IQ.^66^ Changes in the white matter subnetwork connecting parietal, limbic, temporal and occipital areas were significantly associated with IQ, and the white matter subnetwork connecting the nodes, right lingual gyrus left superior parietal gyrus and right cuneus cortex, were associated with processing speed.^69,70^ Fractional anisotropy in the midbody of corpus callosum, the cingulum and the superior longitudinal fasciculus were correlated with general communication composite scores in children cooled for HIE.^64^ Similarly, brain regions involved in attention and visuospatial processing including the precuneus, thalamus, left superior parietal gyrus and left inferior temporal gyrus showed lower connectivity in children cooled for HIE than controls.^70^

## Lack of benefit of therapeutic hypothermia in low- and middle-income countries (LMICs)

By contrast with the clear benefit of TH in high-income countries, the Hypothermia for moderate or severe neonatal encephalopathy in low and middle-income countries (HELIX) trial found that TH had no overall effect on death or disability in infancy, and was associated with an apparent increase in the risk of death.^71^ It is possible that this is partly because only 8% of the babies in the hypothermia group and 13% in the normothermia group in the HELIX trial had a sentinel event, compared with 25% or more in studies from HICs.^72,73^ The presence of clinical seizures before initiation of hypothermia and the predominance of white matter injury compared to basal ganglia and thalamic injury on MRI suggested that these infants were likely exposed to subacute HI starting well before birth.^71^

Infants born after sentinel events may have better outcomes after TH, suggesting that those infants exposed to an acute hypoxic-ischemic insult may benefit more than the infants exposed to subacute hypoxic-ischemia. For example, in a retrospective single-center study of 182 neonates with HIE treated with hypothermia, those who had acute sentinel events had less severe injury on MRI and better motor and language outcomes at 18–36 months of age.^74^ Thus, it is plausible that, in part, the low rate of sentinel events in the HELIX trial may have contributed to the lack of benefit after hypothermia.

Consistent with these findings, a recent case-control study of the whole blood transcriptome of term and near-term infants with HIE from Italy and in the HELIX trial,^75^ found that infants with adverse outcomes despite TH for HIE in high-income settings showed downregulation of eukaryotic translation initiation factor 2, consistent with acute hypoxia. By contrast, in low- and middle-income settings, infants with adverse outcomes expressed aldosterone signaling, which has been associated with intermittent and chronic hypoxia. Although the HELIX trial was not powered to analyze the effect of sentinel events on outcomes after hypothermia, taken together, these data raise the speculative possibility that it might be possible to target treatment to the subset of babies who have had sentinel events in the LMIC.

## Dysglycemia in hypoxic-ischemic encephalopathy

In studies of infants who underwent TH for HIE hypoglycemia, hyperglycemia, and glycemic lability were common. For example, in a secondary analysis of the CoolCap RCT of TH vs normothermia for HIE (*n* = 234 infants), hypoglycemia (OR: 6.2, 95% CI 1.4–27.3), and hyperglycemia (OR:2.7, 95% CI 1.5–4.9) were associated with death and/or severe neurodevelopmental disability at 18 months compared with normoglycemia, independent of TH and severity of HIE.^76^ Further, hypoglycemia, but not hyperglycemia, was associated with multiorgan impairment compared with normoglycemia; however, the comparison was not adjusted for confounders.^77^ More recently, in the HEAL trial (*n* = 491 infants), hypoglycemia was associated with increased risk of death (OR: 2.85, 95% CI 1.09 to7.43) and neurodevelopment impairment (OR:2.50, 95% CI 1.09–7.43), while hyperglycemia was associated with increased risk of death (OR:2.52, 95% CI 1.10 to 5.77) but not neurodevelopment impairment, independent of the severity of asphyxia, encephalopathy and illness and treatment with erythropoietin.^78^

Consistent with these findings, meta-analysis of studies of infants treated with TH (*n* = 446 infants) reported that hypoglycemia was associated with increased risk of abnormal neonatal brain imaging (OR: 1.863, 95% CI: 1.18 to 2.83), especially white matter injury, and death or neuro disability (*n* = 501 infants, OR: 2.17, 95% CI: 1.36 to 3.45).^79^ In two cohort studies (*n* = 428 infants), hyperglycemia increased the odds of abnormal brain imaging compared with normoglycemia (OR:2.41, 95% CI: 1.63 to 3.56), particularly the odds of basal ganglia, thalami, and posterior limb of the internal capsule injury (OR: 2.91 to 3.49) was higher than the white matter or cortical injury (OR: 2.0 to 2.07). Further, hyperglycemia was also associated with higher odds of death or neurodevelopmental impairment at 18 months of age in 5 cohort studies (*n* = 515 infants, OR: 2.74, 95% CI:1.49 to 5.04). Similarly, in the Long-Term Outcome of Neonatal Hypoxic Encephalopathy in the Era of Neuroprotective Treatment with Hypothermia (LyTONEPAL) cohort study of 545 patients with moderate-to-severe HIE, hyperglycemia was associated with greater risk of adverse outcomes compared to normoglycemia (aOR 1.81; 95% CI 1.06–3.11).^80^

It is important to consider that intermittent blood sampling could miss episodic glycemic lability. Supporting this speculation, in a prospective cohort of 108 neonates with NE, intermittent glucose testing missed 50% of hypo- and hyperglycemic events on the first day after birth compared with continuous glucose monitoring (CGM).^79^ This study also showed that a CGM value of > 10.1 mmol/L during the first 48 h after birth was associated with basal ganglia (53% positive predictive value and 91% negative predictive value) and watershed injury (47% positive predictive value and 96% negative predictive value). Importantly, although hyperglycemia > 10.1 mmol/L during the first 48 h after birth was associated with cerebral palsy and developmental scores at 18 months including neuromotor score and Child Behavior Checklist internalizing scores, these associations were not independent of the brain injury patterns on the neonatal MRI. Smaller cohort studies (n = 44 to 47 infants cooled for HIE), showed an association between duration of both hypo (OR 7.1, 95% CI 1.8–20.3) and hyperglycemia (OR 5.4, 95% CI 1.6–15.7) and death or moderate or severe disability at 18–24 months of age,^81^ and occurrence (OR 26.55, 95% CI 2.02–348.5), duration (OR 2.11, 95% CI 1.08–4.14) and greater area under the curve (OR 1.80, 95% CI 1.11–2.91) of hypoglycemia was associated with death and brain injury.^82^ Controlled studies of tight glycemic control are now essential to determine whether dysglycemia is the cause of brain injury or just an association.

## Intracranial hemorrhage and HIE

In the original RCTs of TH versus normothermia for HIE, the incidence of intracranial hemorrhage was 39% and 33% in cooled and non-cooled infants at 8 days of age, predominantly subdural hemorrhage (30%), and 7% and 8% respectively, at 14–16 days of age.^83,84^ More recently, in a retrospective population-based study of infants with HIE (*n* = 7265), intraventricular hemorrhage (IVH) was reported in 0.9% of infants.^85^ In a prospective cohort study of infants cooled for HIE (*n* = 160), the incidence of IVH was 9% (95% CI 5.3–15.0%) and occurred frequently in infants with coagulopathy during TH.^86^ However, the magnitude of hemorrhage was usually small, and not significantly associated with neurodevelopmental outcomes at 18 to 26 months of age (OR:1.24, 95% CI 0.14 to 10.65).

## Interactions between genetics and HIE

In the HEAL trial (*n* = 500 infants), 5% (95% CI 3% to 7%) had genetic or congenital anomalies. Both were associated with a higher risk of death or neurodevelopmental impairment (75% vs 50%, *p* = 0.02) and cerebral palsy (32% vs 13%, *p* = 0.02) compared with the rest of the cohort.^87^ The mechanism of this association is unknown but could reflect either greater risk of HI or vulnerability to HI injury. Supporting the latter possibility, in a cohort study of children with CP, 387 / 1578 (24.5%) had a genetic diagnosis; the subgroup with CP associated with HIE had a significantly greater proportion of genetic diagnoses compared with CP related to other causes, suggesting the possibility of a genetic vulnerability to HIE.^88^ Further, a small cohort of 55 infants with HI suggested that gene-gene interaction in inflammation pathways seemed to be associated with risk of epilepsy after HIE.^89^

## Infection and neonatal encephalopathy

Acute perinatal and postnatal infection can lead to NE with abnormal tone, apnea, and risk of death or neurodevelopmental problems,^90,91^ and acute severe infection can present very similarly to HIE, with hypotonia, acidosis, and seizures. In the UK, prospectively collected data from an infection surveillance network of 30 intensive care units, found that from 2005 to 2014 there were 2171 cases,^92^ for an overall rate of bacterial infection of 6.1/1000 live births. The incidence of both early-onset sepsis and late-onset ( > 48 h) sepsis fell over this period. The majority of cases (76%) were late onset, and sepsis was more common in preterm infants (84%) and low birth weight infants ( < 2500 g, 81%). Although many different pathogens were seen, the most common in early-onset sepsis were group B streptococci and *Escherichia coli*, while *Staphylococcus aureus* and coagulase-negative staphylococci were most common in late onset sepsis.

There is growing evidence that neonatal infection remains highly associated with adverse outcomes. In a meta-analysis of 24 studies involving 121,645 infants (101,657 preterm and 19,988 term),^93^ sepsis was associated with increased risk of cognitive delay (adjusted OR, aOR 1.14 (95% CI: 1.01–1.28)), visual impairment (aOR 2.57 (95%CI: 1.14– 5.82)), hearing impairment (aOR 1.70 (95% CI: 1.02–2.81)) and cerebral palsy (aOR 2.48 (95% CI: 1.03–5.99)). Interestingly, a registry-based study of children with cerebral palsy found that 192 full-term infants with neonatal infection were more likely to have spastic triplegia or quadriplegia (OR 2.4),^90^ whereas infection in preterm infants was not associated with a specific phenotype.

## Pathophysiology of NE related to infection

Multiple experimental studies have confirmed that acute exposure to the Gram negative cell wall component lipopolysaccharide (LPS) leads to a robust inflammatory response and adverse neurological outcomes.^94,95^ In turn, early initiation of anti-inflammatory therapies such as Interleukin-1 blockade improves EEG activity and markedly reduces microgliosis and oligodendrocyte loss.^95,96^ The key knowledge gap is the striking lack of information on whether treatment started after a clinically realistic delay would improve outcomes.^97^

## Interactions of infection with HIE

It is important to appreciate that there is a higher incidence of both early neonatal infection and chorioamnionitis and fetal vasculitis in term infants with HIE.^98^ For example, in the Children’s Hospital Neonatal Database, of 1534 infants with HIE treated with TH, 2.3% had confirmed and 16.6% had suspected infection, far more than the population estimate of 0.5–1.0/1000 term or near-term live births.^99^ Further, in a subset of the HEAL trial, nearly 40% (124/321) had evidence of histological chorioamnionitis.^100^ This is consistent with a previous cohort study in term infants with HIE that found that diffuse chronic villitis was associated with abnormal neurodevelopmental outcomes after TH (OR, 9.29; 95% confidence interval, 1.11–77.73).^101^

## Efficacy of therapeutic hypothermia for inflammation-sensitized hypoxia-ischemia

Overall, preclinical studies support concerns that early onset gram-negative inflammation can worsen outcomes after TH. In P7 rats exposed to systemic LPS injection 4 h before HI, LPS increased brain area loss, apoptosis, microgliosis, and astrogliosis compared with vehicle-HI controls, and treatment with hypothermia did not ameliorate these effects.^102^ Similarly, in LPS-sensitized HI in newborn piglets (human term-equivalent) TH for 14 or 24 h did not improve acute EEG recovery, MRS parameters, or cell survival.^103,104^ In contrast, in P7 rats, gram-positive bacterial mimetics given to induce inflammatory sensitization 8 h before HI, hypothermia was still neuroprotective,^105^ suggesting that hypothermia may still be effective after exposure to gram-positive bacterial infections. Note that these studies used short, sub-optimal durations of TH (14–24 h). Longer, clinical protocols of TH might be more efficacious.

Clinically, it remains unclear whether infants with NE exposed to proven infection or risk factors for early-onset neonatal infection in high-income countries do have worse outcomes. For example, group B streptococcal infection in infants with NE was associated with higher mortality than those with NE alone.^106^ However, in a population-based study from England and Wales (*n* = 5206), in infants treated with TH, the presence of risk factors for early neonatal sepsis such as prolonged rupture of membranes, maternal group B streptococcus, pyrexia or intrapartum antibiotics, and NE, did not increase the risk of death or short-term adverse outcome.^93^ Interestingly, exposure to chorioamnionitis in infants with NE was associated with reduced risk of moderate-severe brain injury and adverse cognitive outcomes,^107^ whereas exposure to chronic villitis was associated with impaired neurodevelopment, suggesting that the precise timing and nature of exposure critically modulates its effects.

## Neonatal stroke

Perinatal arterial ischemic stroke (PAIS) occurs in 1 per 2000–3000 livebirths,^108^, and rarely may be confounded with HIE.^109^ In the HEAL RCT (*n* = 500), PAIS was seen in just 21/470 (4%) of infants who had high-quality MRI scans.^110^ Most infants with PAIS do not meet the criteria for HIE. Maternal fever, prolonged rupture of membranes, prolonged second stage, tight nuchal cord, and failed ventouse delivery were more common in PAIS, while thick meconium, sentinel events, and shoulder dystocia were more common in infants with HIE.^111^ Importantly, infants with PAIS develop seizures significantly later ( ≥ 12 h of age, OR 39.7.4; 95% CI 7.3, 217.0) and seizures are more likely to be unilateral clonic motor jerks (OR 13,4; 95% CI 2.1, 87.9) than in infants with HIE,^112^ with corresponding asymmetry in background activity and sleep-wake cycling.

Cerebral sinus venous thrombosis (CSVT) can mimic HIE but again tends to have much later onset of symptoms at a median age of 3 days (IQR 1–7).^113^ Infants with CSVT may present with seizures, coagulopathy, or persistent thrombocytopenia. Risk factors for CSVT include sepsis/infection and dehydration. Cranial ultrasound including color Doppler may suggest the diagnosis when an intraventricular hemorrhage (IVH) with thalamic extension is seen with lack of flow across one or more of the sinuses.^114^ MRI including MR venography is required to confirm the diagnosis and anticoagulation therapy can be considered as it may reduce the risk of thrombus propagation.^115^ Detailed discussion of the management of PAIS and CSVT and seizure detection can be found in the AHA/ASA Scientific statement on management of stroke in neonates and children.^116^

## CNS malformations

Infants with severe malformations of the CNS seldom present with NE.^117^ More often they present with micro- or macrocephaly, hypertonia, hypotonia, and respiratory problems. A thorough neurological examination is mandatory and may show dysmorphisms and neurocutaneous lesions suggestive of a diagnosis. Severe CNS malformations, like alobar prosencephaly (abnormal separation of the cerebral hemispheres or lobes) and lissencephaly (smooth brain), are most often recognized on early fetal ultrasound and termination of the pregnancy will be considered. Other malformations, like cortical migrational disorder (CMD), will be harder or even impossible to detect on fetal ultrasound or MRI that are usually acquired around 20 weeks’ gestation, as migration~ and cortical folding are not complete. In infants with respiratory problems malformations of the brain stem (brainstem disconnection), kinking of the brainstem in the dystroglycanopathy / Walker-Warburg syndrome or elongated superior cerebellar peduncles, “molar tooth sign” in Joubert syndrome (ciliopathy) can be recognized on both pre- and postnatal MRI.^118,119^

Once abnormalities are recognized on fetal ultrasound, and preferably neurosonography (transvaginal rather than transabdominal ultrasound), additional neuro-imaging with fetal MRI is recommended. This provides more detailed information and in one study additional information was provided affecting prognosis and/or counseling in 18.1% of cases compared to ultrasound,^120^ although again CMD might still be difficult to diagnose with certainty early in the second trimester. Interestingly, MRI found more abnormalities in bilateral than unilateral ventriculomegaly (OR: 4.37, 95% CI 1.21–15.76; p = 0.04). MRI, especially when performed postnatally, identified associated CMD in about 10% of the cases with agenesis of the corpus callosum.^121^ Finally, genetic testing using whole exome sequencing (WES) or whole genome sequencing also plays an important role in fetal ventriculomegaly.^122^

## Neurometabolic disorders

**Fig. 6:**
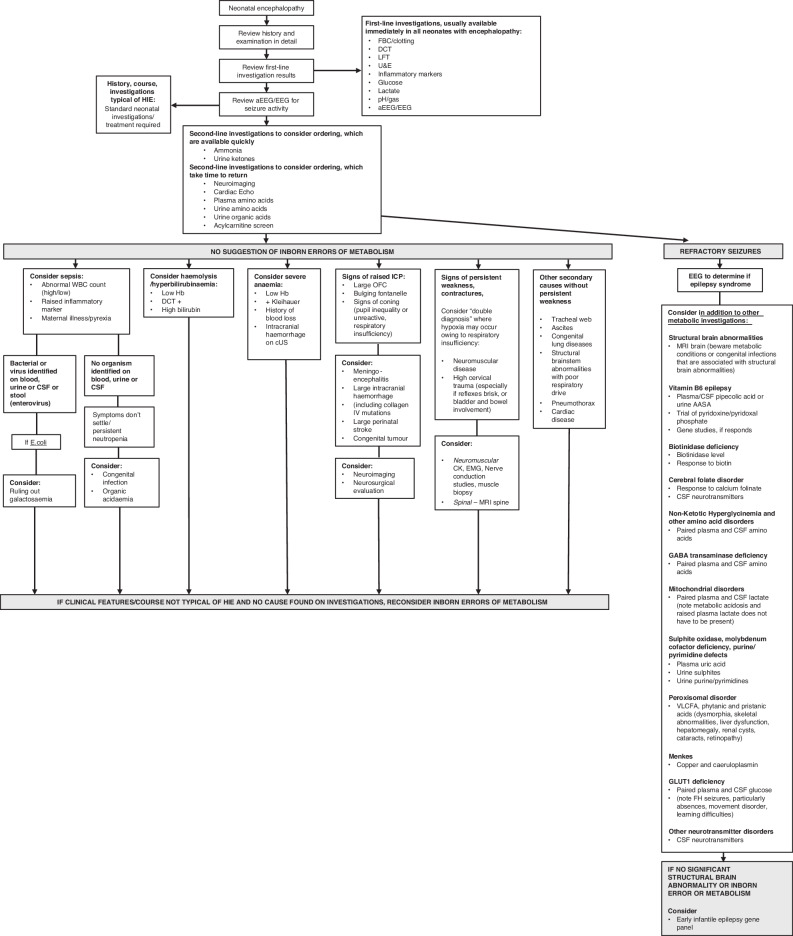
Diagnostic algorithm for NE proposed by Martinello et al.^5^. Creative Commons Attribution (CC BY 4.0) license.

Some inborn errors of metabolism can present soon after birth with NE including seizures, such as urea cycle disorders (ammonia, plasma amino acids, and urine organic acids), non-ketotic hyperglycinemia (cerebrospinal fluid (CSF): plasma glycine), Molybdenum co-factor deficiency (low plasma uric acid & elevated urine s-sulphocysteine),^123^ sulfite oxidase deficiency (elevated urine s-sulphocysteine)^124^ and biotinidase deficiency (reduced biotinidase activity).^125^ A systematic approach in evaluating newborn infants with encephalopathy and suspected inborn errors of metabolism includes clinical examination for dysmorphic features, head size, liver involvement, and cardiac and eye abnormalities,^126^ plus assessment of key biochemical investigations such as metabolic acidosis, lactate, glucose, organic acids, serum ammonia, plasma free fatty acids, and urinary ketones (Fig. 6).

## Genetic and epileptic encephalopathies

### Genetic encephalopathy

One of the major underdiagnosed types of NE is genetic encephalopathy. A retrospective cohort study of 193 cases of NE that was not due to HIE reported a diagnostic yield of 28/193 patients,^127^ many without dysmorphic features or associated congenital anomalies. Diagnosis was best made by exome sequencing. Prader - Willi was the single most common of the non-HIE causes of NE presenting with hypotonia. Similarly, in a retrospective cohort study of 28 infants cooled for HIE who had no known risk factors for perinatal asphyxia, genetic testing including microarrays, whole-exome sequencing, and comprehensive epilepsy panel identified ten patients with channelopathies (*KCNQ2* and *SCN9A* mutation), inborn errors of metabolism (e.g., pyruvate dehydrogenase deficiency, *MTFMT* gene) and other potential candidate genes (e.g., *CDKL5, ISY1, KIF1A*).^128^

A large single center cohort study from China of 366 neonates with NE as part of the Chinese Newborn Genome Project between 2015–17 found on whole exome sequencing that 43 patients (11.7%) had pathogenic or likely pathogenic variants and 10 patients (2.7%) had variants of unknown significance. Neonates with genetic findings had a significantly higher incidence of seizures (96.2%, *P* = 0.0009), but a lower frequency of abnormal MRI (*P* < 0.0001).^129^ Epileptic encephalopathy related genes accounted for nearly half (46.4%) of all genetic defects of NE with seizures.

Other etiologies that can include a genetic basis are vascular conditions like stroke (Col4a1 and Col4a2), neuromuscular disorders like myotonic dystrophy, malformations like tubulinopathies, lissencephaly, holoprosencephaly, dystroglycanopathies and Dandy - Walker and Joubert syndromes. Metabolic syndromes encompass a broad array of disorders presenting with NE and can be diagnosed with appropriate laboratory studies like amino acids and organic acids and acylcarnitine profiling.^7^ NE can be multifactorial with overlap among the different causes. Therefore, all neonates with NE should be evaluated for infection, toxin exposure, seizures, and other forms of acute brain injury like trauma and stroke with appropriate laboratory studies, video EEG, and MRI.^117^

## Genetic epileptic encephalopathies

As discussed above, nearly half of genetic etiologies of NE occur in patients who have seizures without HI. The epileptic encephalopathies include Early Infantile Epileptic Encephalopathy (EIEE), Early Myoclonic Epileptic Encephalopathy, KCNQ2 Encephalopathy, DEND syndrome (developmental delay, epilepsy, neonatal diabetes) and Epilepsy of infancy with Migrating Focal Seizures. Many of these conditions have distinctive EEG patterns, but metabolic studies, neonatal epilepsy panels, and whole exome sequencing are often needed.^130,131^

## NE and risk factors in pregnancy and labor

In unmatched case-control studies, multiple pregnancy and intrapartum factors were associated with NE, after adjusting for confounders, including hypertension in pregnancy (OR: 3.77, 95% CI 1.49 to 9.55), augmentation of labor (OR: 2.23, 95% CI 1.17 to 4.23), no fetal monitoring (OR 2.75, 95% CI 1.71 to 4.22), acute intrapartum events (OR 8.74, 95% CI 1.70 to 45.02) and obstructed labor (OR 3.8, 95% CI 1.96 to 7.36).^106^ Pyrexia in labor may also be associated with risk of NE (OR 6.3, 95% CI 2.7 to 14.8), independent of parity, induction of labor, sex, or receiving epidural anesthesia.^132^ Infants exposed to drugs consumed by their mothers, which were either prescribed or illicit may present with abnormal neurological signs and encephalopathy. Exposure to maternal intake of drugs such as tricyclic antidepressants, selective serotonin reuptake inhibitors (SSRI), antipsychotic medications, and mood stabilizers such as lithium may lead to abnormal tone, poor feeding, somnolence, and decreased deep tendon reflexes.^7^ Exposure to recreational drugs such as amphetamines, opiates, benzodiazepines, cocaine, and barbiturates can be associated with neonatal abstinence syndromes, with high-pitched cry, hypertonia, impaired feeding, and sleep. Infants with less convincing history of perinatal asphyxia and hypertonia, hyperreflexia persisting beyond 48 h after birth should be screened for exposure to possible toxins. Speculatively, exposure to perinatal asphyxia and toxins could contribute to neonatal encephalopathy in combination.

## Diagnostic algorithm for NE

The reader may wish to consult the approach to evaluating the infant with NE from Martinello et al.^5^ (Fig. 6).

### Can we reduce the overall risk of neonatal encephalopathy?

Low- and middle-income countries have a higher burden of NE secondary to birth asphyxia and antenatal factors such as socio-economic deprivation, undernutrition, and sub-optimal antenatal and intrapartum care, and chorioamnionitis increase the risk of NE.^133^ Institutional deliveries, better intrapartum care and support to pregnant women during labor may decrease the incidence of HI. Mathematical modeling data from 184 countries between 1990 and 2010 suggested that the incidence of NE decreased from 11.7 to 8.5 per 1000 live births, secondary to increased institutionalized deliveries and better intrapartum care.^13^ It will be challenging to further reduce the incidence of NE. A meta-analysis of continuous intrapartum cardiotocography with computer analysis compared with visual analysis of cardiotocography (*n* = 54,492 participants) did not reduce the risk of fetal metabolic acidosis.^134^ Nevertheless, there is evidence that artificial intelligence may be able to better utilize the fetal heart rate pattern to identify fetuses who are at high risk of developing acidosis or encephalopathy.^135^

## Conclusions

In sum, recent evidence supports that thorough investigation of the protean causes of NE can lead to more accurate diagnosis and so potentially support our goal of providing the right treatment for the right condition. As discussed in this review, it is now well established that brain injury after both HI and infection/inflammation, and likely other types of NE evolve over time and that interventions need to be matched both specific etiologies and to the precise timing after the insult. Positively, it is now clear that current criteria for HIE are reliable, such that only a small minority have alternative or additional diagnoses. By contrast, infection-related brain injury evolves relatively slowly compared to HIE but a definitive diagnosis of sepsis is often delayed, and it is unknown how late is too late for the proposed interventions. Thus, we now need to find ways to establish the etiology and the timing of injury rapidly after birth. It is likely that a combination of history, examination, neurophysiology and rapid biochemical biomarkers, and genetic analysis, backed by modern imaging, will ultimately let us design novel therapeutics to target the specific diagnosis and timing. The use of these new therapeutics will more accurately be applied individually with the refinements of artificial intelligence, allowing true precision medicine for NE.
